# Effects of Long-Term Controlled-Release Urea on Soil Greenhouse Gas Emissions in an Open-Field Lettuce System

**DOI:** 10.3390/plants13081071

**Published:** 2024-04-10

**Authors:** Xuexia Wang, Bing Cao, Yapeng Zhou, Meng Zhao, Yanhua Chen, Jiajia Zhang, Jiachen Wang, Lina Liang

**Affiliations:** 1 Institute of Plant Nutrition, Resources and Environment, Beijing Academy of Agriculture and Forestry Sciences, Beijing 100097, China; wxx0427@163.com (X.W.); caobing@baafs.net.cn (B.C.); 82101181137@caas.cn (M.Z.); yhchen55@126.com (Y.C.); zhangjiajia_91@163.com (J.Z.); 2 Beijing Engineering Technology Research Center for Slow, Controlled-Release Fertilizer, Beijing 100097, China; 3 College of Land and Resources, Hebei Agricultural University, Baoding 071001, China; zhouyp@hebau.edu.cn

**Keywords:** N_2_O emissions, CO_2_ emissions, controlled-release fertilizer, DCD, lettuce yield

## Abstract

Controlled-release urea (CRU) fertilizers are widely used in agricultural production to reduce conventional nitrogen (N) fertilization-induced agricultural greenhouse gas emissions (GHGs) and improve N use efficiency (NUE). However, the long-term effects of different CRU fertilizers on GHGs and crop yields in vegetable fields remain relatively unexplored. This study investigated the variations in GHG emissions at four growth stages of lettuce in the spring and autumn seasons based on a five-year field experiment in the North China Plain. Four treatments were setup: CK (without N application), U (conventional urea—N application), ON (20% reduction in urea—N application), CRU (20% reduction in polyurethane-coated urea without topdressing), and DCRU (20% reduction in polyurethane-coated urea containing dicyandiamide [DCD] without topdressing). The results show that N application treatments significantly increased the GHG emissions and the lettuce yield and net yield, and DCRU exhibited the lowest N_2_O and CO_2_ emissions, the highest lettuce yield and net yield, and the highest lettuce N content of the N application treatments. When compared to U, the N_2_O emission peak under CRU and DCRU treatments was notably decreased and delayed, and their average N_2_O emission fluxes were significantly reduced by 10.20–20.72% and 17.51–29.35%, respectively, leading to a significant reduction in mean cumulative N_2_O emissions during the 2017–2021 period. When compared to U, the CO_2_ fluxes of DCRU significantly decreased by 8.0–16.54% in the seedling period, and mean cumulative CO_2_ emission decreased by 9.28%. Moreover, compared to U, the global warming potential (GWP) and greenhouse gas intensity (GHGI) of the DCRU treatment was significantly alleviated by 9.02–17.13% and 16.68–20.36%, respectively. Compared to U, the N content of lettuce under DCRU was significantly increased by 6.48–17.25%, and the lettuce net yield was also significantly increased by 5.41–7.71%. These observations indicated that the simple and efficient N management strategy to strike a balance between enhancing lettuce yields and reduce GHG emissions in open-field lettuce fields could be obtained by applying controlled-release urea containing DCD without topdressing.

## 1. Introduction

As an important part of the terrestrial ecosystem, agroecosystems are a major source of global greenhouse gas (GHG) emissions [1], including nitrous oxide (N_2_O), methane (CH_4_), and carbon dioxide (CO_2_), accounting for 14–17% of global directly anthropogenic GHG emissions [2,3,4]. The contribution of these GHGs depends on their global warming potentials and atmospheric lifespans. Specifically, CO_2_ is the leading contributor to climate change, while non-CO_2_ GHGs (N_2_O and CH_4_) also play a crucial role despite their lower emissions. N_2_O has 298 times the global warming potential of CO_2_ based on a 100-year timescale [2,5]. GHG emissions from croplands depend on both environmental variations associated with climate and soil type and agricultural management such as tillage, fertilization and irrigation [6,7]. The excessive use of nitrogen (N) fertilizer has improved crop yields [8], but has also become the largest contributor of GHGs from farmland, accounting for more than 25% of total global N_2_O emissions [9,10]. Hence, there exists an urgent need to develop optimized N fertilization strategies that can mitigate GHG emissions without detrimental impacts on crop yields [5,11].

Many studies have suggested that controlled-release urea (CRU), a newly developed long-acting N fertilizer, could be adopted as an optimal N management practice to improve N use efficiency (NUE), increase crop yields, and reduce GHG emissions. CRU can better match fertilizer nutrient release with crop uptake without additional topdressing [10,12,13]. Previous meta-analyses indicated that CRU could be applied once as basal fertilizer with no effect on grain yields while reducing reactive N losses by 49% worldwide [14], thus decreasing the use of N fertilizer and promoting time- and labor-saving crop production [15,16]. The advantageous effects of CRU on grain yield and NUE depend largely on the synchronization of the N release rate with the N requirements of crops [10]. The application of CRU slows down the release of available N through its physical coating, influences soil NH_4_^+^-N and NO_3_^−^-N content and C/N ratio [17], and alters the structure and activity of the soil microbial community [18]. This may affect soil C and N cycling and then alter CO_2_ and/or reduce N_2_O emissions [12,18]. Although the coated material can control the release rate of nutrients, it cannot control the transformation of dissolved urea in soil, thereby still causing some N losses [13]. 

The combined application of an nitrification inhibitor (NI) and urea is another way to reduce N_2_O emissions, which can control N transformation in soil and improve the NUE [19,20,21]. Dicyandiamide (DCD) is the most widely used NI with relatively low volatility. Previous studies demonstrated that DCD reduced the N_2_O emission by suppressing nitrification and denitrification in soil [19,22,23,24]. Additionally, a three-year field experiment indicated that the application of DCD reduced CO_2_ emissions by 7% [25]. Ahmed et al. [3] reported that urea applied with 1.5% DCD decreased the CO_2_ and N_2_O emissions in soil by 22–172% and up to 94-fold, respectively, compared with those under conventional N fertilizer application. However, the performance and stability of DCD and the transformation of dissolved urea released from CRU vary with soil properties such as soil temperature, pH, and water content [26,27]. Fan et al. [19] confirmed that the CRU amended with 3,4-dimethylpyrazole phosphate (DMPP) could reduce the GHG emissions of open-field vegetables, and that the mitigation effect was made more significant by combining CRU with DMPP. Ge et al. [27] confirmed that the application of CRU and DCD led to significant improvement in wheat yield and NUE, the effect of which was more pronounced under the synergistic use of resin-coated urea and NI.

Vegetable production usually involves intensive fertilization with high N loss potential and contributes to 9% of global cropland N_2_O emissions [28]. China is the largest producer of vegetables in the world, accounting for 41% of total global production [29]. Intensive vegetable production systems take up 13% of the Chinese crop planting area and consume 25% of the national production of chemical fertilizers while emitting 35% of crop-sourced GHGs due to the excessive N (inorganic and organic) inputs, multicropping, and frequent irrigation [30,31]. Therefore, it is imperative to develop appropriate strategies to mitigate GHG emissions from vegetable fields. Some studies have indicated that optimizing N fertilizer management could significantly reduce GHG emissions and maintain the high vegetable yields of Chinese vegetable production systems [13,31]. However, the combined effects of CRU and DCD and the effects of coated urea containing DCD on the GHG emissions from vegetable production systems remain unknown. Lettuce is widely planted in northern China, and mainly cultivated in open fields during spring and autumn. The impacts of optimized N fertilizer management on GHG emissions from open-field lettuce production demand in-depth exploration.

Therefore, a five-year field experiment was conducted to investigate the GHG emissions from an open-field lettuce system fertilized with two types of CRU in northern China. The objectives of this study were to: (1) investigate the long-term effects of different N fertilization treatments on GHG emissions (i.e., N_2_O and CO_2_), soil available N contents, lettuce yield, and N uptake; (2) elucidate the underlying mechanisms and provide guidance for local farmers to optimize N fertilization strategies for the sustainable production of open-field vegetables.

## 2. Results

### 2.1. N_2_O and CO_2_ Emissions

**Figure 1:**
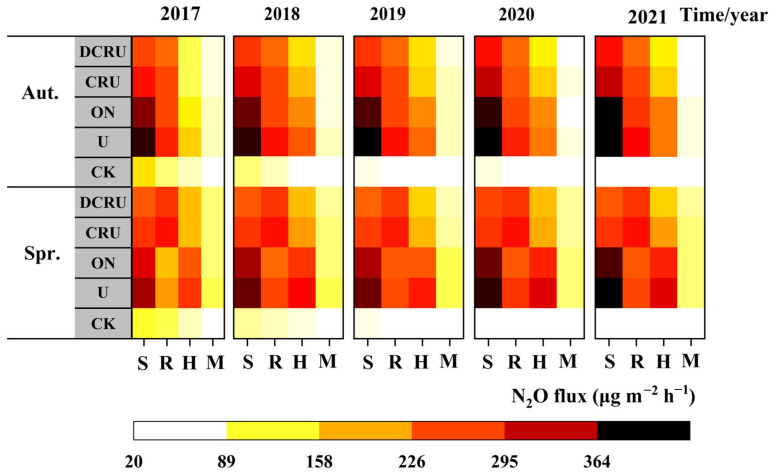
Soil N_2_O emission flux during the experimental period (2017 to 2021) under different treatments. (Note: S—seedling, L—lotus, H—heading, M—mature, Spr.—spring, Aut.—autumn). CK, without N application; U, according to the local farmers’ practice; ON, conventional urea at a reduced 20% N rate; CRU, polyurethane-coated urea at a reduced 20% N rate; DCRU, polyurethane-coated urea containing DCD at a reduced 20% N rate.

Compared with CK, soil N_2_O emission fluxes increased significantly in N application treatments. The N fertilization treatment significantly affected soil N_2_O emission fluxes from lettuce soil, without significant inter-annual or inter-seasonal differences (Table 1). From 2017 to 2021, the average N_2_O fluxes were 169.34–231.12 μg m^−2^ h^−1^ under different N fertilization treatments, with a trend as follows: U > ON > CRU > DCRU. Compared with U and ON, the N_2_O fluxes of DCRU in spring were significantly reduced by 17.51–29.35% (*p* < 0.05) and 7.81–19.81% (*p* > 0.05). Those of CRU were reduced by 10.20–20.72% (*p* > 0.05) and −0.36–8.40% (*p* > 0.05), respectively. The N_2_O fluxes of DCRU in autumn were significantly reduced by 25.81–28.62% (*p* < 0.05) and 14.68–19.81% (*p* > 0.05). Those of CRU were reduced by 17.28–19.83% and 5.33–9.44% (*p* > 0.05), respectively (Table 1). The average N_2_O fluxes reached their lowest level during the mature period, and those in spring were higher than autumn (Figure 1). However, the N_2_O fluxes of U and ON were at their highest at the seedling stage, with those in autumn higher than in spring. The average N_2_O fluxes of CRU and DCRU at the lotus stage were higher than those of other periods in spring, similar to the seedling stage in autumn. The results indicate that the long-term use of controlled-release fertilizers had a significant effect on N_2_O reduction without significant inter-annual or inter-seasonal differences.

**Figure 2:**
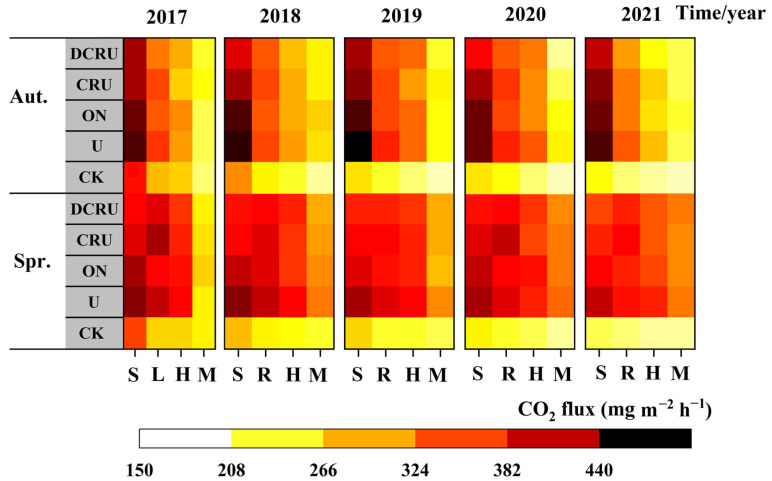
Soil CO_2_ emission flux during the experimental period (2017–2021) under different treatments. Note: S—seedling, L—lotus, H—heading, M—mature, Spr.—spring, Aut.—autumn. CK, without N application; U, according to the local farmers’ practice; ON, conventional urea at a reduced 20% N rate; CRU, polyurethane-coated urea at a reduced 20% N rate; DCRU, polyurethane-coated urea containing DCD at a reduced 20% N rate.

Compared with CK, soil CO_2_ emission fluxes were significantly increased in the N application treatments. The CRU and DCRU reduced the CO_2_ emission fluxes from lettuce soil (*p* > 0.05); however, there were no significant differences between different years and seasons (Table 2). In 2017 to 2021, the average CO_2_ fluxes were 347.99–362.82 mg m^−2^ h^−1^ under different N fertilization treatments during the autumn, i.e., lower than those of the spring (Table 2). The average CO_2_ fluxes gradually decreased from the seedling to the harvest stage in autumn, and those of CRU and DCRU at the lotus stage were higher than those of other periods in spring. The CO_2_ fluxes from seedlings in autumn was higher than that in spring (Figure 2). During the seedling period, compared with U, the CO_2_ fluxes of DCRU were reduced by 8.0–16.54% (Figure 2). The results indicate that the long-term use of DCRU fertilizer could effectively reduce CO_2_ emissions.

**Figure 3:**
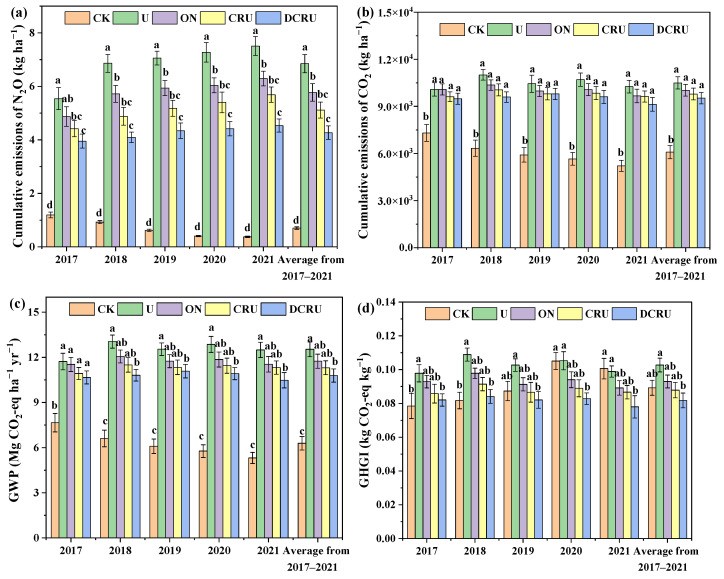
The mean cumulative emissions of N_2_O (**a**) and CO_2_ (**b**), GWP (**c**) and GHGI (**d**) during experimental period (2017–2021) under different treatments. Note: Means with the same lower-case letter across treatments within each figure are not significantly different at *p* < 0.05. The error bars represent the standard error. CK, without N application; U, according to local farmers’ practice; ON, conventional urea at a reduced 20% N rate; CRU, polyurethane-coated urea at a reduced 20% N rate; DCRU, polyurethane-coated urea containing DCD at a reduced 20% N rate.

The cumulative emissions of N_2_O and CO_2_ were consistent with the patterns of N_2_O and CO_2_ fluxes (Figure 3a,b). The cumulative emissions of N_2_O and CO_2_ were 3.95–7.51 kg ha^−1^and 9127.18–11003.84 kg ha^−1^ in the N application treatments, respectively, and decreased under CRU and DCRU treatments in the 2017–2021 period, achieving the lowest values under DCRU. Notably, compared with U, the cumulative emissions of N_2_O from ON, CRU, and DCRU were significantly reduced by 15.70%, 25.34%, and 37.68% (*p* < 0.05), and CO_2_ by 4.46%, 6.77%, and 9.28% (*p* > 0.05), respectively (Figure 3a,b). The GWP was 10.66–13.05 Mg CO_2_-eq ha^−1^ yr^−1^ in this research, compared to U, which decreased by 1.62–7.82%, 6.76–11.85%, and 9.02–17.13% under ON, CRU, and DCRU, respectively, and the GWP of DCRU was significantly reduced by 13.91% (*p* < 0.05) from 2017 to 2021 (Figure 3c). The GHGI was 0.078–0.109 in this research, compared to U, which decreased by 5.06–11.10%, 9.47–17.79%, and 16.68–20.17% in ON, CRU, and DCRU, respectively. Compared with U, the GHGI of DCRU from 2017 to 2021 was significantly reduced by 20.36% (*p* < 0.05) (Figure 3d). The results indicate that the long-term use of DCRU had a significant mitigation effect on the cumulative N_2_O emissions.

### 2.2. Soil NH_4_^+^-N and NO_3_^−^-N Contents

**Figure 4:**
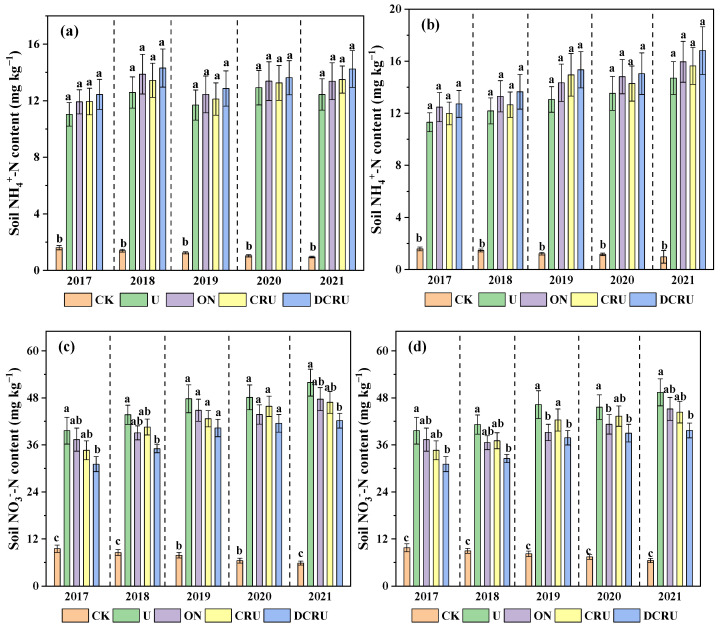
Soil NH_4_^+^-N ((**a**), spring; (**b**), autumn) and NO_3_^−^-N ((**c**), spring; (**d**), autumn) contents during the experimental period (2017–2021) under different treatments. Note: Means with the same lower-case letter across treatments within each figure are not significantly different at *p* < 0.05. The error bars represent the standard error. CK, without N application; U, according to the local farmers’ practice; ON, conventional urea at a reduced 20% N rate; CRU, polyurethane-coated urea at a reduced 20% N rate; DCRU, polyurethane-coated urea containing DCD at a reduced 20% N rate.

Compared with CK, the average soil NH_4_^+^-N and NO_3_^−^-N content was significantly increased in N application treatments. The average soil NH_4_^+^-N content of N application treatments was 10.42–15.23 mg kg^−1^ in spring and 11.38–16.82 mg kg^−1^ in autumn during the lettuce growing seasons in 2017 to 2021; compared with U, those of ON, CRU, and DCRU were increased by 3.62–10.31%, 2.64–14.47%, and 5.48–17.42%, respectively (Figure 4a,b). The average soil NO_3_^−^-N content of N application treatments was 31.07–49.25 mg kg^−1^ in spring and 32.57–51.90 mg kg^−1^ in autumn; compared with U, those of ON, CRU, and DCRU were reduced by 5.77–15.34%, 4.75–12.65% (*p* > 0.05), and 13.78–21.59% (*p* < 0.05), respectively (Figure 4c,d). These results indicated that the long-term use of DCRU increased soil NH_4_^+^-N and reduced NO_3_^−^-N content.

### 2.3. Lettuce Yield and N Content

**Figure 5:**
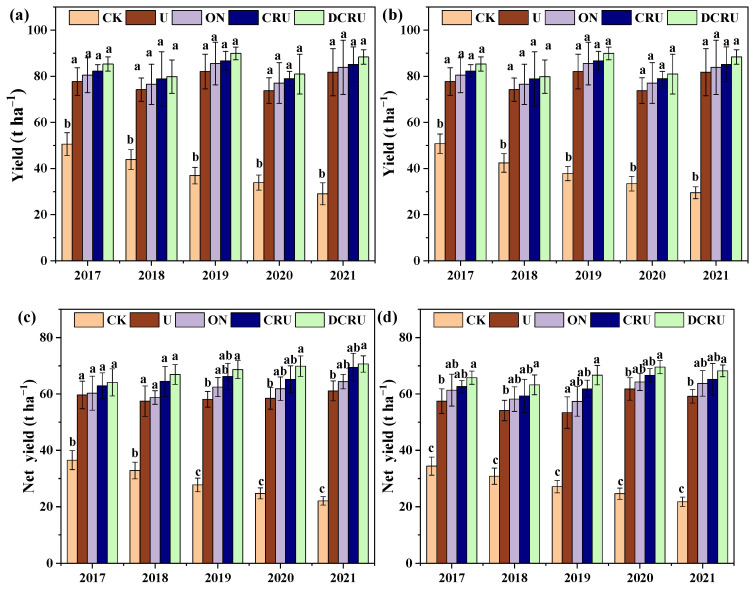
The lettuce yield ((**a**), spring; (**b**), autumn) and net yield ((**c**), spring; (**d**), autumn) during the experimental period (2017 to 2021) under different treatments. Note: Means with the same lower-case letter across treatments within each figure are not significantly different at *p* < 0.05. The error bars represent the standard error. CK, without N application; U, according to the local farmers’ practice; ON, conventional urea at a reduced 20% N rate; CRU, polyurethane-coated urea at a reduced 20% N rate; DCRU, polyurethane-coated urea containing DCD at a reduced 20% N rate.

Compared with CK, the lettuce yield and net yield was significantly increased in N application treatments (Figure 5a–d). Compared with CK, the lettuce yield of ON, CRU and DCRU treatments slightly increased with no significant differences (*p* > 0.05) (Figure 5a,b). Compared with U, the net yield of DCRU was significantly increased by 7.47%, 5.83%, and 5.41% (*p* < 0.05) during the spring season in 2019, 2020, and 2021, respectively, and by 6.79%, 7.48%, 7.35%, and 7.71% during the autumn season in 2017, 2018, 2019, and 2021, respectively (Figure 5c,d). The results indicated that the long-term use of DCRU fertilizer increased the net yield.

**Figure 6:**
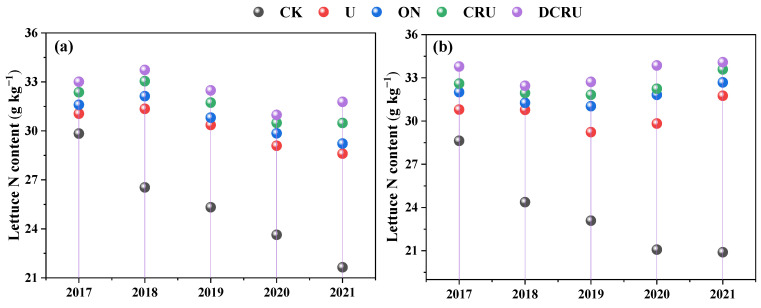
Lettuce N contents in spring (**a**) and autumn (**b**) during the experimental period (2017–2021) under different treatments. Note: CK, without N application; U, according to the local farmers’ practice; ON, conventional urea at a reduced 20% N rate; CRU, polyurethane-coated urea at a reduced 20% N rate; DCRU, polyurethane-coated urea containing DCD at a reduced 20% N rate.

Compared with CK, the N content of lettuce was significantly increased in N application treatments (Figure 6a,b). The N content of lettuce showed no significant differences across different N treatments, seasons, and years (Figure 6a,b). Compared with U, the N content of spring lettuce in ON, CRU, and DCRU was increased by 3.51–4.67%, 5.27–7.63%, and 7.35–12.20%, and that of autumn lettuce was increased by 3.60–8.82%, 3.72–9.96%, and 6.48–17.25%. The results indicated that the long-term use of DCRU could effectively increase the N content of lettuce.

## 3. Discussion

### 3.1. Effects of Different Fertilization Treatments on N_2_O Emissions

The N_2_O in soil originate from the microbial nitrification processes under aerobic conditions and the denitrification processes under anaerobic conditions [32]. The N_2_O emissions are affected by soil inorganic N content, soil temperature and water filled pore space (WFPS) [33,34]. In general, vegetable soils with high chemical N fertilizer inputs and frequent irrigation are major sources of N_2_O [11,34,35]. Wang et al. [31] conducted a meta-analysis focused on Chinese vegetable production systems and found that N_2_O emissions had a positively linear correlation with fertilizer N application rates. Our results were consistent with previous studies. Due to the large amount of N fertilization, the annual N_2_O emissions from the open-field lettuce system reached 7.51 kg ha^−1^ under conventional N application practices (spring and autumn, N 480) (Figure 3a) in this study, which were 1.41 times higher than a wheat–corn rotation system (3.12 kg ha^−1^) in the same region [36]. In this study, cumulative N_2_O emissions were reduced by 15.70% under ON compared to U. Therefore, it is necessary to establish optimized N management strategies in open-field lettuce systems to decrease the N_2_O emissions in this region [7].

Our findings suggest that, compared to CK, the N fertilization treatments significantly affected N_2_O emissions. The CRU significantly decreased the soil N_2_O emissions from lettuce fields compared to conventional N fertilization (Figure 1 and Figure 3), which was in accordance with previous findings [10,14,37]. The soil NH_4_^+^-N and NO_3_^−^-N levels were significantly lower under CRU than ON, possibly because conventional urea dissolved rapidly in the soil solution upon entering the soil. This rapid dissolution leads to a short-term increase in soil N content, reducing N uptake due to limited lettuce growth. As a result, greater accumulation of extra N substrates promotes the nitrification and denitrification processes, ultimately leading to increased N_2_O production [38,39,40]. In our study, the polyurethane film as the outer layer of CRU prevented water penetration into the fertilizer, thereby slowing down the release of N, promoting lettuce uptake of mineral N from the soil, increasing N fertilizer utilization and lettuce yield, reducing substrate N concentrations required for N_2_O formation, and suppressing soil nitrification and denitrification, ultimately resulting in decreased N_2_O emissions [36,41,42]. Except for the high N_2_O fluxes under traditional urea fertilization in 2017–2021 due to the high soil NO_3_^−^-N contents, the N_2_O fluxes of the seedling stage (basal fertilization) were higher than those of the mature period. This was possibly because the basal fertilizer was applied with 40% of the total N inputs, and the lower N requirement for lettuce growth during the seedling stage, resulting in higher soil NH_4_^+^-N and NO_3_^−^-N contents during the seedling stage than the mature period, thus stimulating N_2_O emissions and leading to higher N_2_O fluxes than those from the mature period. Moreover, high concentration of NO_3_^−^ may also inhibit the reduction of soil N_2_O to N_2_, which thus increases the soil N_2_O emissions under higher WFPS [43].

Our study found that the DCRU fertilizer outperformed other treatments in mitigating N_2_O emissions from the open-field lettuce system, possibly due to the synergistic effects of NIs and CRU fertilizers. Previous findings showed that NI amendments are an effective approach to decrease direct soil N_2_O emissions [19,21,44]. The inhibition of the abundance of nitrifiers by NIs directly reduced the nitrification rate, and lowered the soil NO_3_^−^ availability as the substrate for denitrification [13], leading to a reduction in direct N_2_O emissions. Our study also found that compared with conventional N fertilization, the DCRU treatment decreased soil NO_3_^−^-N by 13.78–21.59%, which probably led to the decrease in N_2_O (17.51–29.35%).

In addition to fertilization, other factors such as the irrigation amount [30] and soil properties [11] have also been reported to significantly affect N_2_O emissions in vegetable production systems. Our study found that frequent irrigation and precipitation during the two lettuce seasons (May and June in spring and August and September in autumn) resulted in high soil WFPS (50–73%) and temperature (mean: 13.28–24.58 °C) (Table S1). This was beneficial to N_2_O generation, which was mostly produced via denitrification and oxidizer-denitrification [30,34]. The N_2_O fluxes during the seedling stage in autumn were higher than those in the spring season (Figure 1), due to higher soil temperature and moisture during the seedling stage in autumn and the stimulated soil microbial activity involved in the N cycle, subsequently promoting soil N_2_O production [35]. CRU controls the release rate of N based on soil moisture and temperature [45]. The N_2_O fluxes of CRU and DCRU at the lotus stage in spring and the seedling stage in autumn were higher than those of the other periods, which was possibly because the soil moisture and temperature at this stage favored the rapid release of N and enhanced the N substrate for N_2_O production.

### 3.2. Effects of Different Fertilization Treatments on CO_2_ Emissions

The results of this study indicated that N fertilization practices imposed a significant impact on CO_2_ emissions, with the highest being observed from conventional N fertilization. Our results are consistent with previous studies [46,47]. Huang et al. [48] found that CO_2_ emissions under urea fertilized treatments were two-fold higher compared to non-fertilized treatments. Zhong et al. [47] showed that soil CO_2_ emissions in farmland ecosystems increased with the N application rate. Lin et al. [49] found that, over six growing seasons, the mean growing season soil C emissions were increased by 11.6–29.7% under N fertilization. This could be due to the fact that moderate N application promoted crop root growth, soil microbial activity, and the decomposition of soil organic matter, and consequently increased soil CO_2_ emissions [50]. Another reason is that the decomposition of urea in soil by urease can directly generate CO_2_, further increasing CO_2_ emissions from urea application treatments [51,52]. However, the CO_2_ emission factor from urea for warm and cold cropping seasons in soils in the North Plain of China (the research area) was still unclear, which need to be further explored.

The lowest CO_2_ emissions were observed under the DCRU treatment, except for CK treatment. This is consistent with previous studies reporting that reduced N fertilizer and CRU application had a positive effect on CO_2_ reduction [52,53]. DCD application partially inhibited soil CO_2_ emission [44]. The results of a 3-year field study showed that the application of DCD decreased CO_2_ emissions by 7% [25]. Another study reported that DCD minimized CO_2_ emissions from acidic soils [54]. Similarly, Raza et al. [26] also stated that DCD significantly decreased CO_2_ emissions from calcareous soils. However, few studies have reported the mechanism of DCD effects on soil respiration and mineralization, which needs to be varied further under field conditions.

Soil CO_2_ is mainly produced by crop root respiration and decomposition of organic matter by soil microorganisms, which is affected by soil temperature and moisture content [55]. Our results showed slightly higher CO_2_ emissions in spring than in autumn, and this can be associated with higher soil temperature and stronger microbial activity in spring than in autumn [56]. The highest CO_2_ fluxes were observed in the seedling stage, except those of CRU and DCRU at the lotus stage in spring. This may have resulted from the altered soil nutrient availability and carbon supply under N fertilizer application [57]. After basal fertilization, the chemical N fertilizer application increased the contents of inorganic N in soil, and provided a sufficient N source for microorganisms, thereby increasing the soil CO_2_ emissions in the seedling period. At the lotus stage in spring, CRU and DCRU released more N due to the higher soil temperature, and provided sufficient N for soil microbial activities, which could be attributed to increased soil CO_2_. This study observed that the CO_2_ fluxes from the seedling stage in autumn were higher than those in spring. A possible reason may be that, in the seedling stage, the higher temperature and WFPS of the soil promote microbial respiration and decomposition activity, and increased the rate of CO_2_ produced by urea decomposition in the autumn over that in spring.

### 3.3. Responses of Lettuce Yields and GHGI to N Application

Within a certain range of N fertilizer application rates, the crop yields and quality increased with increasing N inputs [58]. Nevertheless, excessive N application could not improve yield, and caused low N fertilizer utilization efficiency, which not only wastes resources but also reduces soil quality [59]. These results were supported by our study, showing that neither reducing N inputs nor applying CRU fertilizer led to a reduction in lettuce yield. The previous studies confirmed that CRU had the potential to balance wheat yield and NUE by promoting plant N uptake and utilization due to the rational supply of nutrients, without decreasing grain productivity compared with conventional urea, even in soils with high fertility [60,61]. Similar to previous studies, our results also showed that CRU simultaneously improved lettuce yield and net yield compared with conventional urea at the same N rate. In contrast to CRU, urea dissolved rapidly into the soil after fertilization, resulting in the loss of a large amount of N that failed to be absorbed by plants in time, and making it difficult for plants to absorb sufficient N for growth at the middle and late growth stages, thus decreasing lettuce yield and N use efficiency [34]. The CRU may synchronize N release with crop demand compared to traditional fertilizers [4] and can better meet the crop nutrient requirements and improve plant N uptake (Figure 6), contributing greatly to lettuce N accumulation and yield [61]. Notably, our results indicated that the net yield could be significantly increased under long-term use of DCRU fertilizers by 5.41–7.71%, due to the simultaneous limitation of N fertilizer release and inhibition of nitrification in soil. Therefore, DCRU application is a simple and efficient N management strategy to obtain target vegetable yields and economic benefits while increasing NUE and alleviating environmental risks. However, the effect of DCRU on the NUE and yields with changes in soil inorganic N content were unclear, and it is worth exploring in the following experiment.

The mean GWP decreased with N application rate, but the inhibitory effect under ON was much less than that of CRU and DCRU (Figure 4). In comparison with conventional urea, DCRU significantly decreased the GWP. The decrease in GWP under CRU and DCRU was mainly due to the reduced N_2_O emissions, which was in accordance with previous studies [16,18,27]. Therefore, the reasonable application of DCRU may be an important measure for slowing down GHG emissions from vegetable systems in the future.

The GHGI is a comprehensive indicator of the greenhouse effect and the economic benefits of farmland [31,62,63]. We found that compared with conventional urea, CRU and DCRU decreased GHGI. Meanwhile, GHGI was found to decrease by CRU in the previous study [10]. Therefore, the trade-offs between crop yields and GHG emissions in vegetable fields could be obtained by controlled-release fertilizer combined with DCD.

This study provided a scientific basis for promoting environmentally friendly, low-cost, and efficient fertilization practices to achieve global C neutrality. However, to date, our understanding of the effects of the fertilizer coating materials on soil and plants and the long-term effects of different fertilization methods is still insufficient. Future research should take various soil properties and agricultural practices into consideration, and investigate the long-term effects of controlled-release N fertilizer combined with DCD on soil and crops.

## 4. Materials and Methods

### 4.1. Study Site

A five-year experiment was conducted from 2017 to 2021 at Yongsheng Garden agricultural planting center (116°41′32″ E, 39°41′2″ N) located in Tongzhou District in the suburb of Beijing in northern China. It has a temperate continental monsoon climate, with an average annual air temperature of 11.3 °C and an average annual precipitation of 620 mm (mostly occurring in July and August). The soil at the experimental site is classified as sandy soil (sand 55.8%, clay 34.9%, silt 9.3%) according to the Chinese Soil Taxonomy System. A 6 yr wheat–maize rotation was carried out prior to this experiment. The soil characteristics (0–20 cm) are listed as follows: pH 8.12, organic carbon 1.58 g kg^−1^, NH_4_^+^-N 1.53 mg kg^−1^, NO_3_^−^-N 9.80 mg kg^−1^, total nitrogen 1.90 g kg^−1^.

### 4.2. Experimental Design

Four different N fertilizer management treatments were established: (1) CK: without N fertilizer; (2) U: conventional urea fertilizer (46% N, according to local farmers’ practice) applied at 300 kg N ha^−1^; (3) ON: conventional urea fertilizer applied at 240 kg N ha^−1^; (4) CRU: polyurethane-coated urea (42% N, 60-day release, developed by the Institute of Plant Nutrition, Resources and Environment at the Beijing Academy of Agriculture and Forestry Sciences) at 240 kg N ha^−1^; (5) DCRU: polyurethane-coated urea containing DCD (DCD mixed with urea was coated by polyurethane after granulation, DCD:N = 1:100) at 240 kg N ha^−1^.

All treatments were arranged in a randomized complete block design with four replicates. The area of each plot was 28 m^2^ (7 m × 4 m). A ridge with 0.3 m width and 0.3 m height was set up to prevent the exchange of water and nutrients between plots. The open field was planted with the lettuce (*Lactuca sativa*) ‘Sheshou No. 101’, which is a common local cultivar with a growth period of approximately 60 days in the spring and autumn. The lettuce seedlings were transplanted on 15 April or 18 April and 19 August or 23 August in the spring and autumn, respectively.

For the U and ON treatments, 40%, 20%, 20%, and 20% of the total N was applied as basal, first, second, and third topdressing fertilizers at the seedling, lotus, and heading stages, respectively. For the CRU and DCRU treatments, the total N was applied all at once as basal fertilization. For all treatments, P and K fertilizers were applied at rates of 78 kg P ha^−1^ and 184 kg K ha^−1^, and all the P and K fertilizers were applied as basal fertilization. Basal fertilizer was surface broadcast by hand, whereas the topdressing fertilizers were dissolved and applied with drip irrigation. The irrigation amount during the entire growth period was 945 m^3^ ha^−1^. The U and ON treatments had topdressing irrigation every 10 days, a total of 3 times, with each fertilization irrigation of 120–150 m^3^ ha^−1^, and the CK, CRU, and DCRU treatments were irrigated with equal amounts of clean water. The experiment was repeated for five years (from 2017 to 2021).

### 4.3. Sampling and Measurement

Six fresh plants were randomly collected from each plot at the mature stage, and the fresh lettuce plants were weighed for lettuce yield, then removed the inedible parts and weighed to obtain the net lettuce yield. All lettuce plants were dried in an oven at 70 °C for over 48 h and then weighed for the aboveground dry matter yield. The N content of aboveground lettuce biomass was determined using the Kjeldahl digestion method.

Six soil samples (0–20 cm) were randomly collected from each plot each time at the seedling (on the 6th day after basal fertilizer), rosette (on the 5th day after first topdressing), heading (on the 5th day after third topdressing), and mature stages during the growing season of the lettuce using a stainless-steel soil sampling auger (diameter 4.5 cm). The collected soil samples in the same plot were homogeneously mixed into a composite sample for further chemical analysis. The CRU particles were picked out to ensure that the soil does not contain fertilizer particles.

The soil pH was measured by a potentiometer (Delite B1020, Beijing, China) using a water-to-soil ratio of 5:1. Soil organic carbon (SOC) and total N was determined using an elemental analyzer (Flash Smart NC SOIL, Beijing, China). Soil NO_3_^−^-N and NH_4_^+^-N were extracted by dissolving 20 g of fresh soil with 100 mL of 1 mol L^−1^ KCl solution. The soil extracts were then colorimetrically detected for NO_3_^−^-N and NH_4_^+^-N contents using a continuous flow injection analyzer (Auto Analytic 3, Seal Analytical, Germany).

The soil emissions of N_2_O and CO_2_ were measured by the static closed-chamber technique in the seedling, rosette, heading, and mature stages. The chamber consists of a plexiglass base collar (50 cm L × 40 cm W × 20 cm H) and a removable top chamber (50 cm L × 40 cm W × 50 cm H) equipped with a battery driven 12 V fan at the center of its inner top. The gas measurements were performed between the lettuce plants. Throughout the lettuce growing season in each year, the sampling was carried out in the morning between 9:00 am and 11:00 am every 2 days for 8 days during the base fertilizer period, every three days after every topdressing N fertilization or irrigation event for 10 days, or once every 7 days otherwise. Four gas samples were successively collected per plot at an interval of 10 min (0, 10, 20, and 30 min) from the chamber headspace using a 25-mL gas-tight syringe, then immediately transferred to 12 mL air-evacuated gas-tight glass vials. The samples were analyzed by using a gas chromatograph (Agilent, HP7890, Agilent, Santa Clara, USA) equipped with an electron capture detector (ECD) to detect N_2_O and a flame ionization detector (FID) to detect CO_2_ [64]. The CO_2_ and N_2_O exchange fluxes were calculated using Equation (1) [65]:

(1) $$F=\rho \times h\times \frac{∆c}{∆t}\times \frac{273}{273+T}$$

where *F* in CO_2_ is mg m^−2^ h^−1^ and N_2_O is μg m^−2^ h^−1^, *ρ* is the concentration of N_2_O and CO_2_ (e.g., N_2_O: 1.977 kg m^−3^, CO_2_: 1.997 kg m^−3^). *h* is the height of the effective space in the chamber (m), ∆c is the gas concentration difference, ∆t is the time interval, and *T* is the mean soil temperature for each sampling (°C).

The cumulative emissions of CO_2_ and N_2_O were calculated for each treatment according to Equation (2) [31]:

(2) $$\;CGE=\sum_{i=1}^{n}(V_{i+1}+V_{i})/2\times (t_{i+1}-t_{i})\times 24$$

where *CGE* is the cumulative emission (kg ha^−1^) for each gas, *n* is the total sampling time, *V_i_* and *V_i+_*_1_ are the measured fluxes of two consecutive sampling days, and (*t_i+_*_1_ − *t_i_*) is the time interval between two adjacent sampling days.

The warming potentials (GWP) of N_2_O are 298 times that of CO_2_, on a 100-year timescale [2]; therefore, the equation for calculating the GWP (kg CO_2_-eq ha^−1^) is Equation (3):

GWP = GCO_2_ + 298GN_2_O (3)

where GCO_2_ (kg CO_2_ ha^−1^) and GN_2_O (kg N_2_O ha^−1^) are the cumulative CO_2_ and N_2_O emissions, respectively.

Greenhouse gas intensity (GHGI) refers to the comprehensive warming potential per unit of output, and is calculated according to Equation (4):

GHGI = GWP/Y (4)

where GHGI is the GHG emission intensity (kg CO_2_-eq kg^−1^ lettuce yield), GWP is the total amount of greenhouse gas emissions (kg CO_2_ eq ha^−1^), Y is the yield of lettuce (kg ha^−1^).

### 4.4. Statistical Analysis

Statistical analysis was performed using SPSS 24.0 (IBM Co., New York, NY, USA). Before the analysis, a Shapiro–Wilk test and Levene test were employed to check the normality and variance homogeneity of the data. One-way analysis of variance (ANOVA) was performed to assess the effects of different treatments on lettuce yield, N uptake, soil NH_4_^+^-N and NO_3_^−^-N content, and N_2_O emissions. The means of significant effects were compared using Duncan’s multiple range test (*p* < 0.05). All the figures were drawn with Origin 2023 (OriginLab Co., Northampton, MA, USA).

## 5. Conclusions

In comparison with conventional urea, N_2_O emissions reduced but the reduction effect on CO_2_ emissions was not obvious, and lettuce yield and net yield were maintained in ON. CRU was effective in controlling N_2_O by prolonged NH_4_^+^-N availability in the soil of the open-field lettuce system and in improving the N content of lettuce and net yield. DCRU was the most effective in mitigating N_2_O and CO_2_ fluxes and the mean cumulative effect of N_2_O and CO_2_ emissions, with a reduction of up to 37.68% and 9.28%%, respectively, and the net yield was also significantly increased by 5.41–7.71%, due to the improved N absorption of the lettuce in higher soil NH_4_^+^-N, lower NO_3_^−^-N content under the long-term use of DCRU. Therefore, the simple and efficient N management strategy to obtain trade-offs between GHG emissions and lettuce yields in the open-field lettuce system could be obtained by controlled release of fertilizer containing DCD without topdressing. This study provided a theoretical basis for the efficient and sustainable development of open-field vegetable production by adopting optimized N management practices.

## Figures and Tables

**Table 1:** Effect of different treatments on N_2_O emission fluxes (μg m^−2^ h^−1^) in the lettuce growing seasons.

Year	Seasons	CK	U	ON	CRU	DCRU
2017	Spring	60.83 ± 7.66 c	203.29 ± 22.58 a	181.89 ± 27.80 ab	182.55 ± 28.23 ab	167.69 ± 21.21 b
	Autumn	64.44 ± 7.31 c	200.22 ± 24.50 a	178.23 ± 32.56 ab	160.51 ± 31.33 ab	142.92 ± 24.19 b
2018	Spring	42.79 ± 5.71 c	233.69 ± 26.21 a	207.62 ± 31.30 ab	190.18 ± 26.94 ab	169.48 ± 25.88 b
	Autumn	40.87 ± 6.26 c	226.40 ± 30.50 a	197.83 ± 27.90 ab	183.61 ± 28.93 ab	165.17 ± 22.53 b
2019	Spring	22.62 ± 2.04 c	241.27 ± 29.95 a	203.64 ± 30.02 ab	192.25 ± 31.63 ab	172.12 ± 27.54 b
	Autumn	23.30 ± 3.18 c	228.76 ± 28.82 a	199.67 ± 29.15 ab	186.42 ± 30.18 ab	167.78 ± 26.93 b
2020	Spring	16.62 ± 1.77 c	245.31 ± 32.08 a	215.63 ± 28.31 ab	197.23 ± 32.14 ab	175.75 ± 28.89 b
	Autumn	18.31 ± 2.05 c	231.94 ± 28.89 a	200.35 ± 27.31 ab	189.66 ± 30.34 ab	170.95 ± 30.05 b
2021	Spring	15.30 ± 1.75 c	259.57 ± 30.23 a	227.81 ± 35.94 ab	209.14 ± 31.33 ab	183.04 ± 28.23 b
	Autumn	16.22 ± 1.70 c	253.22 ± 31.69 a	221.72 ± 28.55 ab	204.51 ± 30.59 ab	178.95 ± 32.60 b
2017–2021	Spring–autumn	32.13 ± 7.66 c	231.12 ± 27.68 a	202.44 ± 31.83 ab	189.01 ± 30.47 ab	169.34 ± 26.32 b

Note: Values are means ± SE (standard error) of four replicates. The same letter within a row means no significant difference at the level of 0.05. CK, without N application; U, according to the local farmers’ practice; ON, conventional urea at a reduced 20% N rate; CRU, polyurethane-coated urea at a reduced 20% N rate; DCRU, polyurethane-coated urea containing DCD at a reduced 20% N rate.

**Table 2:** Effect of different treatments on CO_2_ emission fluxes (mg m^−2^ h^−1^) in the lettuce growing seasons.

Year	Seasons	CK	U	ON	CRU	DCRU
2017	Spring	251.92 ± 12.86 b	347.99 ± 22.78 a	344.89 ± 18.19 a	340.05 ± 19.77 a	327.16 ± 19.48 a
	Autumn	256.22 ± 20.44 b	311.12 ± 28.38 a	306.29 ± 25.15 a	301.86 ± 25.75 a	295.55 ± 19.82 a
2018	Spring	231.80 ± 6.61 b	362.82 ± 16.91 a	347.17 ± 13.28 a	341.81 ± 12.47 a	333.55 ± 11.40 a
	Autumn	214.90 ± 11.79 b	322.07 ± 20.78 a	314.22 ± 21.50 a	306.16 ± 22.29 a	299.99 ± 18.98 a
2019	Spring	206.35 ± 7.76 b	354.67 ± 13.48 a	337.91 ± 14.42 a	335.03 ± 13.06 a	326.79 ± 22.95 a
	Autumn	200.78 ± 8.30 b	330.01 ± 26.06 a	321.36 ± 22.63 a	312.77 ± 23.07 a	309.25 ± 19.74 a
2020	Spring	191.16 ± 7.66 b	357.43 ± 15.97 a	351.70 ± 10.04 a	343.92 ± 13.29 a	336.76 ± 19.28 a
	Autumn	192.56 ± 6.50 b	331.89 ± 21.33 a	315.24 ± 22.79 a	306.89 ± 24.08 a	295.95 ± 20.53 a
2021	Spring	198.52 ± 7.04 b	349.79 ± 14.64 a	333.88 ± 12.92 a	327.97 ± 19.77 a	323.16 ± 22.52 a
	Autumn	190.31 ± 6.54 b	302.37 ± 25.20 a	295.28 ± 26.72 a	283.10 ± 16.86 a	272.86 ± 18.58 a
2017–2021	Spring–autumn	209.45 ± 10.15 b	337.02 ± 9.95 a	326.79 ± 7.03 a	319.96 ± 7.84 a	312.10 ± 9.53 a

Note: Values are means ± SE (standard error) of four replicates. The same letter within row means no significant difference at the level of 0.05. CK, without N application; U, according to the local farmers’ practice; ON, conventional urea at a reduced 20% N rate; CRU, polyurethane-coated urea at a reduced 20% N rate; DCRU, polyurethane-coated urea containing DCD at a reduced 20% N rate.

## Data Availability

The data presented in this study are available on request from the corresponding author. The data are not publicly available due to the project was not yet completed.

## Supplementary Materials

The following supporting information can be downloaded at: https://www.mdpi.com/article/10.3390/plants13081071/s1, Table S1: Changes in soil temperature, WFPS, and precipitation in the lettuce growing seasons (means ± SE).
