# Supplementary material for: Effects of Long-Term Controlled-Release Urea on Soil Greenhouse Gas Emissions in an Open-Field Lettuce System
Source: Plants (Basel). 2024 Apr 10;13(8):1071. doi: 10.3390/plants13081071 (PMC11054608; doi:10.3390/plants13081071)
Supplement: Supplementary file 1 [file plants-13-01071-s001.zip › plants-2749394-supplementary.pdf]

Supplementary Materials:

Table S1. Changes in soil temperature, WFPS, and precipitation in lettuce growing seasons (means±SE).

Year	Seasons	Index	Seedling	Lotus	Heading	Mature
2017	Spring	Temperature (℃)	13.35±1.61 b	16.28±2.26 ab	18.92±2.74 a	22.28±2.39 a
		WFPS (%)	58.94±6.32 a	63.27±5.24 a	61.32±5.16 a	57.20±5.87 a
		Precipitation (mm)	0	0.65	0.85	0.60
	Autumn	Temperature (℃)	23.21±1.28 a	19.45±1.78 ab	17.64±2.84 b	15.23±2.98 b
		WFPS (%)	67.56±3.76 a	60.21±4.24 ab	59.24±5.01 ab	55.16±5.62 b
		Precipitation (mm)	31.9	20.80	8.30	1.20
2018	Spring	Temperature (℃)	12.38±2.04 b	16.03±2.63 ab	18.31±2.98 a	21.05±3.02 a
		WFPS (%)	60.02±3.97 a	63.24±4.39 a	62.45±4.58 a	58.21±3.98 a
		Precipitation (mm)	0	0	1.00	0.40
	Autumn	Temperature (℃)	24.02±1.56 a	20.23±2.19 ab	18.03±2.75 b	15.65±3.04 b
		WFPS (%)	65.45±2.98 a	61.37±3.67 ab	58.93±4.65 ab	54.69±5.42 b
		Precipitation (mm)	29.8	19.40	10.30	0.90
2019	Spring	Temperature (℃)	14.03±3.01 b	17.21±2.48 ab	19.34±2.33 ab	22.96±2.08 a
		WFPS (%)	60.21±4.89 a	62.56±5.07 a	57.89±4.32 a	53.57±5.26 a
		Precipitation (mm)	0	0.42	0	1.60
	Autumn	Temperature (℃)	23.68±1.67 a	20.88±2.21 ab	17.84±2.67 ab	16.01±2.96 b
		WFPS (%)	66.38±2.89 a	64.04±3.21a	60.56±4.68 a	59.21±5.22 a
		Precipitation (mm)	34.45	21.95	10.20	2.40
2020	Spring	Temperature (℃)	13.89±2.03 b	16.98±2.71 ab	18.55±2.41 a	22.01±2.52 a
		WFPS (%)	56.84±6.42 a	61.89±5.73 a	59.89±5.03 a	55.28±4.89 a
		Precipitation (mm)	0	0	1.20	2.89
	Autumn	Temperature (℃)	23.78±1.56 a	20.87±2.16 ab	19.04±2.86 ab	16.78±3.22 b
		WFPS (%)	64.86±3.07 a	62.01±4.65 a	59.56±5.21 a	56.78±5.77 a
		Precipitation (mm)	23.67	18.24	12.45	3.16
2021	Spring	Temperature (℃)	14.06±3.01 b	16.98±2.45 ab	19.63±2.23 ab	22.57±1.78 a
		WFPS (%)	60.06±4.56 a	62.01±5.32 a	58.95±5.78 a	56.78±5.05 a
		Precipitation (mm)	0.42	0	1.52	1.06
	Autumn	Temperature (℃)	24.02±1.67 a	21.00±1.99 ab	18.95±2.32 ab	17.03±3.02 b
		WFPS (%)	64.24±2.95 a	61.89±3.64 a	60.33±4.85 a	57.97±5.28 a
		Precipitation (mm)	20.67	14.82	13.26	5.41

Daily average temperature and WFPS (water filled pore space) at 10:00-14:00 am during the growth stage. Same letter within row means no significant difference at the level of 0.05.
